# Silenced cutters: mechanisms and effects of protease inhibition in plant–pathogen interactions

**DOI:** 10.1093/jxb/eraf156

**Published:** 2025-04-12

**Authors:** Catarina Paiva-Silva, João Proença Pereira, Frederico Marcolino, Andreia Figueiredo, Rita B Santos

**Affiliations:** Grapevine-Pathogen Systems Lab, Biosystems and Integrative Sciences Institute (BioISI), Faculty of Science, University of Lisbon, Lisboa, Portugal; Grapevine-Pathogen Systems Lab, Biosystems and Integrative Sciences Institute (BioISI), Faculty of Science, University of Lisbon, Lisboa, Portugal; Grapevine-Pathogen Systems Lab, Biosystems and Integrative Sciences Institute (BioISI), Faculty of Science, University of Lisbon, Lisboa, Portugal; Grapevine-Pathogen Systems Lab, Biosystems and Integrative Sciences Institute (BioISI), Faculty of Science, University of Lisbon, Lisboa, Portugal; Grapevine-Pathogen Systems Lab, Biosystems and Integrative Sciences Institute (BioISI), Faculty of Science, University of Lisbon, Lisboa, Portugal; Albert-Ludwigs-Universitat Freiburg, Germany

**Keywords:** Cell death, plant immunity, plant pathogen, proteases, protease inhibitors, signalling

## Abstract

Proteases are essential enzymes in plants that play multiple roles in immunity, including molecular recognition, programmed cell death, and the degradation of pathogen proteins. During plant–pathogen interactions, both organisms have evolved mechanisms to regulate protease activity. Plants produce specific inhibitors to prevent excessive or harmful proteolysis, while pathogens counteract these defences by deploying molecules that block proteases and weaken plant immunity. Despite significant progress in understanding protease function, many regulatory mechanisms remain unexplored. This review examines the roles of endoproteases in plant responses to biotic stress and the diverse strategies employed by both plants and pathogens to modulate their activity. We discuss known protease inhibition mechanisms and highlight emerging methodologies that offer new insights into protease regulation. Additionally, we explore biotechnological applications, including genetic engineering and chemical inhibitors, aimed at enhancing plant resistance to pathogens. By integrating current knowledge with innovative research tools, we can uncover novel protease regulatory pathways and develop new strategies to improve plant resilience. Understanding these mechanisms not only advances fundamental plant biology but also holds potential for sustainable agricultural practices in the face of evolving pathogen threats.

## Teaser

We summarize and integrate current knowledge on protease inhibition in plant–pathogen interactions and speculate on ways in which this could be harnessed to improve plant resilience to biotic stresses.

## Introduction

Plant diseases pose a major threat to agricultural systems. Whether it be through yield losses or lower product quality, diseases can quickly spread if left uncontrolled, jeopardizing food security. In practice, plant diseases are controlled via the use of phytopharmaceuticals, which directly fight or prevent their onset. While effective, this approach presents a well-documented threat to sustainability (Tudi *et al.*, 2021), contributing significantly to pollution in conventional large-scale agriculture and exacerbating the pressing issue of rising pesticide resistance. Therefore, it has become urgent to find new mechanisms, tools, and technologies that make plants more resilient to disease, enabling the mitigation of phytopharmaceutical usage in agriculture.

Understanding how plants and pathogens interact is essential for the development of new sustainable methods of disease control. These interactions are heavily shaped by pathogen lifestyle, which can be categorized as biotrophic, necrotrophic, and hemibiotrophic. While biotrophic pathogens colonize living host tissue and obtain nutrients from it, necrotrophs infect and kill host tissues, deriving nutrients from dead cells. Alternatively, hemibiotrophic pathogens exhibit a two-phase infection process, beginning with a biotrophic phase followed by a necrotrophic stage (Lo Presti *et al.*, 2015). Irrespective of lifestyle, the interaction between plant and pathogen is underpinned by a complex and robust host immune response, consisting of not only physical barriers, but also cellular and molecular pathways. These pathways can be described as two interconnected layers of defence, pattern-triggered immunity (PTI) and effector-triggered immunity (ETI), which have been synthesized by the zig–zag model (Jones and Dangl, 2006). PTI serves as the first line of defence and is activated upon the recognition of conserved pathogen-associated molecular patterns (PAMPs) or microbe-associated molecular patterns (MAMPs) via surface-exposed pattern recognition receptors (PRRs). Since PAMPs are generally conserved across microorganisms, PTI can confer resistance to most non-adapted pathogens and plays an important role in basal immunity during infection (Zipfel, 2014). To evade this defence mechanism, pathogens deploy effector molecules enhancing pathogen virulence, leading to effector-triggered susceptibility (ETS). Resistant hosts can detect this attack and mount a strain-specific immune response that is called ETI, the second line of defence of the plant (Jones and Dangl, 2006; Ngou *et al.*, 2022). These two layers of defence are intrinsically linked, since PTI enhances ETI responses. Likewise, ETI potentiates PTI by increasing the abundance of PTI signalling components (Santos *et al.*, 2020; Ngou *et al.*, 2021; Chang *et al.*, 2022; X. Yu *et al.*, 2024). These interconnected immune responses rely on a complex network of molecular players, including proteases, which are crucial regulators of plant defence.

Proteases hold great importance in a myriad of cellular processes that require protein degradation, whether they be developmental or in response to stressors (van der Hoorn and Rivas, 2018; van der Hoorn and Klemenčič, 2021). Proteases are divided into different classes based on the amino acids present in their active sites, the most common being serine, cysteine, aspartic, and threonine proteases, while metalloproteases have a characteristic metal cation in their active site (Godson and van der Hoorn, 2021). Proteases are involved not only in protein turnover but also in protein activation. They do this by cleaving inhibitory or regulatory domains, which can change the subcellular localization and/or function of a protein (Godson and van der Hoorn, 2021), thereby positioning them as crucial components in signalling cascades (Fernández-Fernández *et al.*, 2023). Proteases are also an integral part of plant defence systems, playing various roles in plant–pathogen interactions beyond cleaving pathogen-derived proteins. In this context, the role of proteases begins in the apoplast, the extracellular space where the surveillance of danger signals and initial pathogen recognition takes place (Del Corpo *et al.*, 2024). Here, proteases can play a role in pathogen recognition by participating in PTI-associated mechanisms, such as the generation of immunopeptides (Chen *et al.*, 2023). They are also key players in the establishment of the hypersensitive response (HR), a form of localized programmed cell death (PCD) that is essential for preventing the spread of the pathogen within the plant (Liu *et al.*, 2024b).

There are several models that explain how plants recognize pathogen effectors. The original Guard Model, based on the gene-for-gene hypothesis, identified specific pairs of resistance (R) proteins and avirulence (Avr) effectors (Jones and Dangl, 2006). However, many of these interactions appear to be indirect. To address this, the model was expanded to propose that R proteins guard one or more host target proteins. When a pathogen effector modifies one of these guarded targets, the R protein detects the change and triggers an immune response, which can explain why a single R protein is sometimes able to recognize multiple effectors. Further evidence that effectors may interact with several host proteins led to the proposal of the Decoy Model. In this model, plants produce decoy proteins that mimic the true targets of effectors. These decoys do not perform the function of the original target but instead lure the effectors into binding, which then inadvertently activates the immune response of the plant (van der Hoorn and Kamoun, 2008; Paulus and van der Hoorn, 2018). For example, Rcr3 (Required for Cladosporium resistance-3), an apoplastic cysteine protease, acts like a co-receptor for Avr2 of the fungal pathogen *Cladosporium fulvum* together with the tomato Cf-2 receptor-like protein (RLP) (Rooney *et al.*, 2005). In contrast, pathogens are also known to employ proteases as pathogenicity mechanisms, ranging from direct host protein and cell wall degradation to evasion of immune mechanisms (Santos and Figueiredo, 2021; Lee Erickson and Schuster, 2024). Apoplastic proteases are also essential for the generation of PAMPs. In Arabidopsis, the apoplastic proteases SBT5.2 and SBT1.7 are crucial for immune defence. They cleave bacterial flagellin to release the flg22 peptide, which triggers pathogen recognition and reactive oxygen species (ROS) production. In mutants lacking these proteases, flg22 generation is delayed, compromising the defence response of the plant (Matsui *et al.*, 2024). Also, pathogen-derived type III effector proteases enter host cells to subvert plant immunity through several mechanisms. They inactivate immune signalling by cleaving key receptors and signalling molecules (Dissmeyer *et al.*, 2018; Ravalin *et al.*, 2019). Additionally, these proteases expose recognition sites on host proteins, marking them for degradation by the ubiquitin–proteasome system (UPS), and some directly disrupt the UPS by cleaving polyubiquitin chains or ubiquitin-like proteins (Pruneda *et al*., 2016; Langin *et al*., 2020; Xiang *et al.*, 2020). Although their inhibitors are unknown, another knowledge gap in the field, these strategies underscore the complex tactics pathogen effectors use to undermine plant defences (Mooney *et al.*, 2021).

Due to the pivotal role of proteases in the immune response on both sides of the interaction, fine-tuning their activity becomes particularly important, avoiding excessive proteolysis which could have several deleterious effects. On the one hand, endogenous regulation via calcium or pH places a checkpoint on endogenous proteolysis in plants (reviewed in Fernández-Fernández *et al.*, 2023). On the other hand, pathogens can modulate protein levels or their subcellular location, adding to the complexity of how plant proteases are regulated, not only endogenously but also by plant pathogens (Huang and van der Hoorn, 2025). Beyond these mechanisms, protease inhibition also holds tremendous importance for plant biotic stress, with mechanisms ranging from inhibition by endogenous plant protease inhibitors (PIs) to the secretion of pathogen-derived inhibitors targeting plant proteases (Jashni *et al.*, 2015; Rawlings *et al.*, 2018). These small proteins are divided into different groups based on their structure, function, or sequence homology (e.g. Bowman–Birk inhibitors, Kunitz inhibitors, Kazal-like inhibitors, cystatins, and serpins, among others] (Grosse‐Holz and van der Hoorn, 2016; Hellinger and Gruber, 2019). An important feature of PIs is the fact that each can inhibit different proteases from different classes, overcoming protease redundancy and possibly altering the outcome of the interaction. This also occurs in the opposite direction, with several inhibitors from different classes being able to inhibit the same protease, making them hubs for inhibition (Grosse‐Holz *et al.*, 2018).

In this review, we summarize the current standing on knowledge of protease inhibition strategies and biological outcomes in the context of plant biotic stress, approaching this question from both sides of the interaction. Additionally, we discuss different biotechnological approaches that can be applied to achieve plants with increased resilience to pathogens and pests.

## Protease inhibition at the plant–pathogen interface

**Fig. 1.**
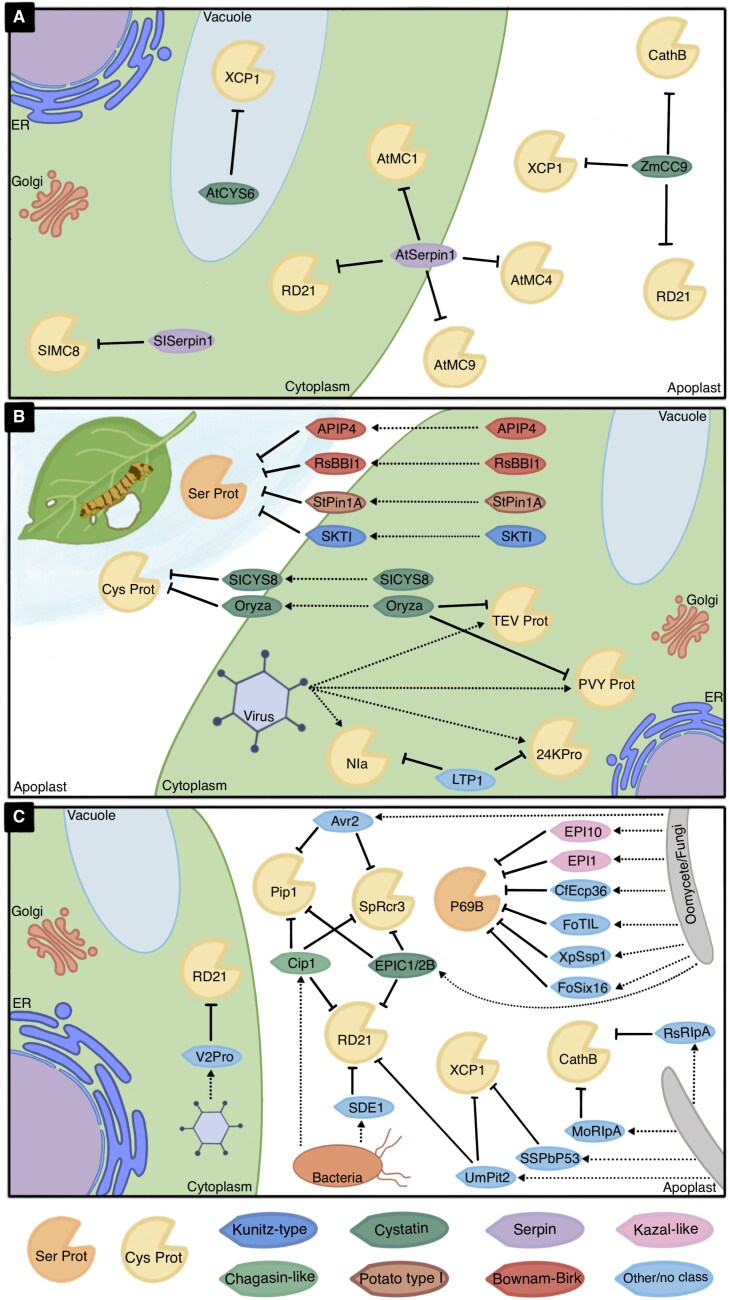
Events of protease inhibition in plant biotic stress at three different levels. (A) Inhibition of endogenous plant proteases by plant protease inhibitors; (B) inhibition of pathogen/pest proteases by plant-derived protease inhibitors; (C) inhibition of plant proteases by pathogen-derived protease inhibitors. All proteases are denoted by the name of their homologue in Arabidopsis unless indicated otherwise. *At*MC1/4/9, Metacaspase-1/4/9; RD21, Resistance to desiccation 21; XCP1, Xylem cysteine protease 1; PVY, potato virus Y; TEV, tobacco etch virus; Rcr3, Required for Cladosporium resistance-3; Pip1, Phytophthora-inhibited protease 1; *At*CYS6, Cystatin 6; *Zm*CC9, Corn cystatin 9; SKTI, serine Kunitz trypsin inhibitor; *Rs*BBI1, Bowman–Birk inhibitor; *St*Pin1A, Serpin1A; LTP1, Lipid transfer protein 1; EPIC1/2B, extracellular protease inhibitors with a cystatin-like domain 1/2B; Cip1, C14-inhibitor protein-1; SDE1, Sec-delivered effectors; Pit2, protein involved in tumors 2; *Cf*Ecp36, extracellular protein 36; *Fo*TIL, trypsin inhibitor-like protein; *Fo*Six16, secreted into xylem 16; *Xp*Ssp1, small secreted peptide 1; RlpA, Rare lipoprotein A.

Due to the prolific roles and diverse locations of protease action in immunity mechanisms, it is important to consider the implications of their inhibition at different levels. Thus, we will analyse the dynamics and functions of protease inhibition at three levels: (i) endogenous mechanisms of inhibition; (ii) inhibition of host proteases by pathogen-derived inhibitors; and (iii) inhibition of pathogen proteases by host-derived inhibitors (Fig. 1).

### Endogenous mechanisms of inhibition

Given the crucial role of proteases in cellular mechanisms, plants regulate their activity through endogenous mechanisms (summarized in Table 1).

**Table 1.** Summary of the discussed protease inhibitor–protease pairs and affected functions

Inhibitor	Target protease	Targeted function	Reference
14-3-3ι	Cys proteases	Cytochrome *c* sequesters 14-3-3ι, which is an inhibitor of caspase-like activity, for HR enactment.	Elena‐Real *et al.* (2021)
*At*Serpin1	*At*MC1	Impairment of HR in response to *P. syringae.*	Lema Asqui *et al.* (2018)
	*At*MC4/9	Inhibition by *At*Serpin1 validated *in silico* and *in vitro*, predicted to occur in the apoplast.	Vercammen *et al.* (2006)
	RD21	Impairment of HR upon inhibition of RD21.	Lampl *et al.* (2013)
*Sl*Serpin1	*Sl*MC8	Impariment of HR. Increased resistance to biotrophs and decreased resistance to necrotrophs.	Basak *et al.* (2024)
*At*CYS6	XCP1	Inhibition of XCP1 by *At*CYS6 blocks RBOHD degradation, maintaining ROS homeostasis. Increased tolerance to necrotrophs and hemibiotrophs.	Liu *et al.* (2024a)
*Zm*CC9	XCP1	*Zm*CC9 inhibits apoplastic Cys proteases which cleave a maize-derived peptide, triggering an immune response.	van der Linde *et al.* (2012); Ziemann *et al.* (2018)
	Cathepsin B		
	RD21		
APIP4	Ser Proteases	The inhibitory potential of APIP4 is reduced by an *M. oryzae* Avr protein and increased by its cognate R protein in rice. APIP4 also stabilizes *Os*PR1-like and is a positive cell death regulator.	Zhang *et al.* (2020, 2024)

A paradigmatic family of plant PIs that has received considerable attention in the context of plant–pathogen interaction is the Serpin family. Serpin proteins were initially identified as serine PIs, although many members are also able to inhibit cysteine proteases with roles in regulation of PCD. For instance, *Arabidopsis thaliana* Serpin1 (AtSerpin1) was shown to inhibit Metacaspase 1 (*At*MC1) (Lema Asqui *et al.*, 2018), and *At*MC4 and *At*MC9 (Vercammen *et al.*, 2006) via a ‘suicide’ irreversible inhibitor mechanism. Interestingly, while the inhibition of *At*MC1 was found to occur in the apoplast, the interactions between *At*MC4 and *At*MC9 were predicted to take place in the apoplast. The interaction between *At*Serpin1 and *At*MC1 was verified *in vivo*, and a possible role for cell death inhibition was also observed in plants overexpressing the PI when infected with the bacterial pathogen *Pseudomonas syringae* pv. *tomato* DC300 (PtoDC3000) (Lema Asqui *et al.*, 2018). Interestingly, serpins were also found to inhibit caspases in animals (Choi *et al.*, 2019), along with other cell death-related proteases such as cathepsin B (CathB; Luke *et al.*, 2022), suggesting a high degree of conservation and assigning to serpins an important function as regulators of PCD. As expected, the inhibition of metacaspases by Serpin1 transcends the model plant Arabidopsis, with *Sl*Serpin1 also having been shown to inhibit *Sl*MC8 in tomato (Basak *et al.*, 2024) in the cytoplasm. SlMC8 was also shown to be a positive regulator of cell death and a modulator of tolerance against pathogens in a lifestyle-specific manner, where overexpression of this protease rendered tomato more tolerant to biotrophs but more susceptible to necrotrophs (Basak *et al.*, 2024). Considering these results across models, it is tempting to speculate that the inhibition of metacaspases by Serpin1 could serve as a tolerance mechanism against necrotrophy by blocking the onset of PCD, providing an essential mechanism of protease self-regulation by the host.

More clues for this hypothesis lie in the inhibition of RD21 (RESPONSIVE TO DESICCATION-21) by AtSerpin1. RD21 is a highly conserved papain-like cysteine protease (PCLP) with prolific roles in various aspects of plant biology, and has been identified as a pro-cell death protease with significance in immune responses (Huang and van der Hoorn, 2025). For instance, Lampl *et al.* (2013) demonstrated that not only do AtSerpin1 and RD21 interact *in vivo* in the cytoplasm, but that this interaction is also formed in association with cell death. In addition, upon exposure to oxalic acid, a known inducer of cell death, both overexpressors of AtSerpin1 and *rd21* mutants showed comparably lower cell death rates, consistent with the hypothesis of inhibition of AtSerpin1 by *rd21*. Interestingly, this same group showed greater tolerance to necrotrophic interactions with *Botrytis cinerea* and *Sclerotina sclerotium*, while becoming more susceptible to infection by *Colletotrichum higgisianum*, a hemibiotroph (Lampl *et al.*, 2013). These results point towards a model where self-inhibition of RD21 by AtSerpin1 enhances plant resilience to necrotrophs, possibly due to an inhibition of cell death, whereas biotrophic pathogens can colonize the hosts tissue more extensively due to reduced PCD. However, the opposite reaction for RD21 has also been reported for a necrotrophic pathogen, where *rd21* mutants were rendered more susceptible to *B. cinerea.* Conversely, the same work noted that both PtoDC3000 and *Hyaloperonospora arabidopsidis*, a biotroph, did not show any differential phenotype in *rd21* mutants, which contrasts with the previously presented results for AtSerpin1 inhibition of RD21 (Shindo *et al.*, 2012). One key distinction between this work and that performed by Lampl and associates was the mode of infection—while the former was conducted on whole plants, the latter was done in detached leaves. This could be of importance, considering the impact of systemic responses and plant integrity, and its effects on infection outcome (Orłowska *et al.*, 2013). Another hypothesis put forth by the authors is that *B. cinerea* does not encode PLCP inhibitors, which would make this pathogen sensitive to RD21 activity. It is important to highlight that these differing outcomes correspond to distinct inhibition mechanisms—with AtSerpin1 exhibiting endogenous inhibition and *B. cinerea* possibly undergoing pathogen-induced inhibition. Consequently, this may lead to varying effects on infection outcomes, emphasizing the critical role of protease inhibition in biotic interactions.

Recently, *A. thaliana* 14-3-3ι, a homologue of a human 14-3-3 protein that is a negative regulator of cell death, was described to potentially act as an inhibitor of caspase 3-like activity, in a dose-dependent way. Simultaneously, cytochrome *c* may act as an inhibitor of 14-3-3ι, possibly playing a role as a pro-cell death factor (Elena‐Real *et al.*, 2021). Indeed, another rice 14-3-3 has also been identified as a negative regulator of cell death, although the precise mechanism was not elucidated (Manosalva *et al.*, 2011). Conversely, the tomato 14-3-3 TFT7 was found to be a positive regulator of PCD, although through interaction with the *Pseudomonas* resistance and fenthion sensitivity/resistance to *Pseudomonas syringae* pv. *tomato* (Prf/Pto) NLR complex and mitogen-activated protein kinase kinase kinase (MAPKKK) upon Avr protein recognition (Sheikh *et al.*, 2023). This suggests a more complex role for 14-3-3 proteins in PCD (reviewed in Sheikh *et al.*, 2024). In animals, 14-3-3 proteins have been reported to both directly and indirectly inhibit apoptosis, either via inhibition of caspase proteins (Kalabova *et al.*, 2020) or through the interaction and sequestering of pro-apoptotic molecular players (Nomura *et al.*, 2003; Elena-Real *et al.*, 2018), respectively. It would thus be important to scrutinize if 14-3-3ι is a direct inhibitor of cell death-related proteases or if it acts upstream of them, rendering it a broader regulator of cell death.

Beyond PCD, proteolysis and protein turnover are also relevant for immune responses (Liu *et al.*, 2024b). For instance, it was recently found that xylem cysteine peptidase 1 (XCP1) is responsible for accelerating the degradation of respiratory burst oxidase homologue D(RBOHD), a homologue of NADPH oxidase, indirectly regulating ROS levels during immune responses. In addition, a cystatin protein, cystatin 6 (CYS6), was found to directly inhibit XCP1 in the vacuole, thus regulating the degradation of RBOHD and, consequentially, maintaining oxidative homeostasis so as to avoid ROS burst. As a result, *cys6* plants were rendered more susceptible to the necrotroph *B. cinerea* and the hemibiotroph *Pseudomonas syringae* pv. *maculicola*, probably because of enhanced ROS levels and cell death, although the latter of these was not directly assessed (Liu *et al.*, 2024a). In line with the regulation of RD21, another cysteine protease, an argument can be made regarding the usefulness of cysteine protease inhibition as a positive regulator of immune responses against necrotrophy, probably associated with the mitigation of cell death. Indeed, XCP1 was recently found to possess caspase-like activity, targeting pathogenesis-related protein 1 (PR1) and producing an immunogenic peptide—CAP-derived peptide 9 (*At*CAPE9)—upon exposure to salicylic acid (SA), being an important process in the establishment of systemic acquired resistance (SAR) (Chen *et al.*, 2023). This is a remarkable case where endogenous (resistance) protein cleavage is conducive to an improved immune response to biotrophs. Interestingly, however, the production of CAPEs from PR1 cleavage was first identified in tomato by Chen *et al.* (2014) in response to both wounding and methyljasmonate (MeJA) treatment. JAs have been linked to SA antagonism, and vice versa, with SA being linked to biotrophy responses and JA to necrotrophy (Caarls *et al.*, 2015), although this notion has come to be disputed in recent times (Ullah *et al.*, 2022). This particularity leads to the question: could the production of CAPEs from the cleavage of PR1 be a conserved, basal mechanism of immunity, irrespective of pathogen lifestyle? If so, it would represent yet another key feature of proteases in plant immunity, making them even more important targets of regulation.

Even though all previous cases of self-regulation of protease activity have been reported to occur in the cytoplasm, these processes can also take place in the apoplast. This is of particular relevance due to the importance of this compartment in plant immune responses, as well as the roles proteases play in the apoplast. An interesting example of this type of inhibition is enacted by the *Zea mays* apoplastic corn cystatin-9 (CC9), an inhibitor of maize apoplastic cysteine proteases that are both regulated by and triggers of SA signalling. CC9 was found to inhibit apoplastic PLCPs such as homologues of RD21, CathB, and XCP1 and, in the process, to be responsible for suppressing SA-induced transcriptional modulation. Accordingly, *cc9* mutants were more tolerant to *Ustilago maydis*, a biotroph, making CC9 a maize susceptibility factor to this pathogen, especially considering that *CC9* is up-regulated upon infection (van der Linde *et al.*, 2012). Despite establishing this important link between PLCP inhibition and SA signalling impairment, the exact mechanism behind it remained undiscovered until Ziemann and colleagues uncovered the missing link, under the form of an immunogenic peptide that is both SA responsive and a trigger of SA accumulation. In this work, the *Z. mays* immune signalling peptide (Zip1) was found to be processed by apoplastic PLCPs from its immature form, ProZip1, and not only promotes the accumulation of SA but also induces similar transcriptional remodelling patterns to those triggered by SA treatment (Ziemann *et al.*, 2018). In this way, CC9 inhibits the formation of Zip1 in the apoplast, hindering SA signalling and, consequentially, the response against biotrophs.

Although self-regulation of protease activity may appear circumscribed to the host, external interference from pathogens can occur, adding another layer of complexity to an already intricate system. Indeed, the case of APIP4, a rice Bowman–Birk inhibitor, is one where the ‘tug-of-war’ of pathogen Avr genes and host R genes meets protease inhibition. APIP4 was shown to possess trypsin inhibition activity both *in vitro* and *in vivo*, with this activity susceptible to being modulated by interaction with rice Piz-t or *Magnaporthe oryzae* AvrPiz-t. Indeed, while the interaction between AvrPiz-t Interacting Protein 4 (APIP4) and AvrPiz-t reduced the trypsin inhibitory ability of APIP4, Piz-t was noted to potentiate not only its trypsin inhibitory function, but also its gene expression and protein accumulation (Zhang *et al.*, 2020). Ultimately, APIP4 was noted to impart increased tolerance to *M. oryzae*, with its knockout rendering plants more susceptible. However, the mechanism by which APIP4 exerts its function as a resistance gene remained unclear, until Zhang *et al.* (2024) uncovered its role in promoting *Os*PR1aL gene expression and stability together with *Os*BBTI5, another Bowman–Birk trypsin inhibitor. Furthermore, when stabilized by both PIs, *Os*PR1aL appeared to confer broad-spectrum resistance against both *M. oryzae* and *Xanthomonas oryzae*, the causal agent of rice leaf blight. Some steps were also taken in understanding what role PCD may play in the observed increased tolerance. Since the triple knockout *apip5apip4osbbti5* presented less pronounced cell death than the single knockout *apip5*, a known negative regulator of cell death (Zhang *et al.*, 2022), a probable role for positive regulation of cell death can be attributed to APIP4. This is an interesting role to ascribe to a PI, since protease inhibition is generally linked to negative cell death regulation. However, it is important to note that the specific targets of inhibition of APIP4 are not known, given that only trypsin inhibition was assessed. It is also interesting to speculate on how this system of broad-spectrum resistance translates into other purely biotrophic or necrotrophic pathogens, since both microorganisms evaluated in this work are hemibiotrophs.

### Inhibition of pest/pathogen proteases by host-derived inhibitors

While the endogenous regulation of protease function via inhibitors is of great relevance, it is also important to consider how both sides of the interaction impact protease function. Indeed, both pathogens and pests use proteases to complete their life cycles, with plants counteracting their activity by producing inhibitors, from viral proteases that ensure capsid formation to insect digestive proteases which are essential for nutrition (summarized in Table 2).

**Table 2.** Summary of the discussed plant-derived protease inhibitors, their target pathogen/pest proteases, and affected functions.

Inhibitor	Target protease	Targeted function	Reference
APIP4	Insect Ser proteases	Inhibition of digestive enzymes in the midguts of pests and restriction of their growth.	Yao *et al*. (2023)
SKTI			Franco *et al.* (2004)
*Rs*BBI1			Mohanraj *et al.* (2018)
*St*Pin1A			Dunse *et al.* (2010)
Oryzacystatin	PVY proteases	Restriction of viral proliferation.	Gutierrez-Campos *et al.* (1999)
	TEV proteases		
LTP1	Nia	LTP1 is translocated to the cytoplasm upon infection, inhibiting Cys proteases of two pathogenic viruses. Restriction of viral proliferation.	Ji *et al.* (2024)
	24KPro		

The current understanding of plant PIs as mechanisms of tolerance against biotic stress is more strongly linked to defence against herbivory, serving as inhibitors of digestive enzymes present in the guts of insect pests. By inhibiting enzymes such as trypsin and chymotrypsin, pests are left with nutritional deficiencies that restrict their growth *in planta* (Divekar *et al.*, 2023). In fact, the gene expression of many plant PIs is induced by herbivory and its quintessential molecular signals such as JA and systemin. Various plant PIs targeting various classes of proteases were shown to impart resistance against different insect pests, such as the soya Kunitz-type inhibitor (SKTI) (Franco *et al.*, 2004), the Bowman–Birke inhibitor1 (*Rs*BBI1) (Mohanraj *et al.*, 2018), and potato-type I and II inhibitors (*St*Pin1A) (Dunse *et al.*, 2010; reviewed in Divekar *et al.*, 2023). These constitute strong deterrents of herbivory and thus have been extensively used in biotechnological approaches. However, due to the high adaptability often observed in pests, insects have developed mechanisms to overcome PI-mediated mechanisms of resistance, from overexpressing targeted proteases to producing other proteases that are insensitive to inhibition by specific PIs (Divekar *et al.*, 2023). This continuous arms race is of great relevance when considering plant PIs as biocontrol measures, as this approach could conceivably be overcome by the deployment of these strategies from plant pests. One such example is the case of NaPI, a potato type II trypsin and chymotrypsin inhibitor that is unable to inhibit chymotrypsin from *Helicoverpa punctigera*, with this need fulfilled by harnessing a potato type I PI (Dunse *et al.*, 2010).

Interestingly, the previously discussed Bowman–Birk PI APIP4 was found to be up-regulated in plants exposed to volatiles emitted by neighbouring plants infested with striped stem borer (SSB) larvae (*Chilo suppressalis*), suggesting that APIP4 may be of importance in the response against this pest (Yao *et al.*, 2023). This was soon validated, since the overexpression of APIP4 also increased rice resilience to SSB larvae, resulting in a decrease in the mass of larvae feeding on plant tissue (Liu *et al.*, 2021). This broad spectrum is remarkable, considering the increased tolerance APIP4 also confers against *X. oryzae* and *M. oryzae*, which would make it a valuable target for crop breeding. It is interesting to speculate whether other PIs have this same broad spectrum of action, considering the ubiquity of proteases as drivers of pest proliferation, pathogenicity, or immunity. For this, it is essential not only to assess the effectiveness of PIs across types of biotic interactions but also to uncover their targets or alternative mechanisms of action.

Plant PIs have also garnered some attention in the context of plant–virus interactions. This is because viral proteases hold great importance in viral replication, and thus proliferation of viral infections, by cleaving the nascent viral polyprotein into mature capsid proteins (Zephyr *et al.*, 2021). Overexpression of some PIs was noted to impart tolerance against various viruses of the Potyvirus genus, a widespread genus of plant pathogenic viruses that carry cysteine protease genes. For instance, overexpression of orzyacystatin, a rice cysteine PI, in tobacco plants rendered them more tolerant to both tobacco etch virus (TEV) and potato virus Y (PVY), members of the Potyvirus genus, while not having a notorious effect on tobacco mosaic virus (TMV) (Gutierrez-Campos *et al.*, 1999). While TMV does not employ cysteine proteases as its processors of the polyprotein precursor, TEV and PVY do, suggesting that oryzacystatin may play a specific role in inhibiting viral cysteine proteases, hindering disease progression.

Recently, one project provided a great deal of insight into protease–inhibitor interaction in the context of plant–virus interactions and provided important information regarding the role of PIs against viral infections. In this study, Ji and associates identified lipid transfer protein 1 (LTP1) of cowpea as a resistance factor against both cowpea mosaic virus (CPMV) and soybean mosaic virus (SMV), restricting their proliferation by inhibiting their respective cysteine proteases, 24KPro and Nia (Ji *et al.*, 2024). This polyvalency is worth noting, since it may constitute a broad-spectrum resistance mechanism against various viruses. Interestingly, the subcellular localization of LTP1 was modulated not only upon CPMV infection, but also by 24KPro itself, where it was shown that both these factors triggered the translocation of LTP1 from the apoplast to the cytoplasm (Ji *et al.*, 2024). This translocation may explain why the *in vivo* 24KPro–LTP1 interaction is only detected at high levels after CPMV infection, revealing an extremely disease-specific mechanism of pathogen-derived protease inhibition by an endogenous inhibitor. This feature may also represent a form of ‘self-defence’ against unwanted protease inhibition, with a comparable example being found in phytaspases, a subset of plant subtilases that are accumulated in their inactive form in the apoplast, only being translocated to the cytosol during PCD (Schaller *et al.*, 2018).

### Inhibition of host proteases by pathogen-derived inhibitors

Since proteases are part of important defence mechanisms, counteracting their function is fundamental for a compatible interaction. To do so, pathogens produce and secrete PIs that directly inhibit plant-derived proteases, thus inhibiting proteolysis or interactions with other proteins (summarized in Table 3).

**Table 3.** Summary of discussed pathogen-derived protease inhibitors, their target host proteases, and affected functions

Inhibitor	Target protease	Targeted function	Reference
EPIC1/2B	RD21	Inhibition of RD21 increases susceptibility to respective pathogen	Kaschani *et al.* (2010)
Cip1			Shindo *et al.* (2016)
SDE1			Clark *et al.* (2018)
V2Pro			Liu *et al.* (2023)
*Mi*CE108			Yu *et al.* (2024b)
*Um*Pit2	RD21	Inhibition of apoplastic Cys proteases impairs SA signaling and tolerance to *U. maydis*	Mueller et al. (2013)
	XCP1		
SSPbP53	XCP1	Inhibition of several apoplastic PLCPs, with differing degrees. May promote tolerance to *Pl. brassicae*	Pérez-López et al. (2021)
*Cf*Ecp36	P69B	Restriction of pathogen growth	Homma et al. (2023)
*Fo*TIL			
*Fo*Six16			
*Xp*Ssp1			
EPI1/10		Restriction of pathogen growth. Inhibition of P69B blocks the production of immunogenic peptides	Tian et al. (2004, 2005); Wang et al. (2021)
*Mo*RlpA	Cathepsin B	Predicted to interact *in silico*	Sarkar et al. (2023)
RsRlpA		Negative regulation of HR. May play a role in the biotrophic stage of *R. solanum*	Charova et al. (2020)
Avr2	Rcr3	Rcr3 acts as a co-receptor of Avr2 with Cf-2. Recognition of Avr2 triggers HR	Rooney et al. (2005)
	Pip1	Inhibition increases susceptibility to *C. fulvum*	Shabab et al. (2008)
EPIC1/2B	Rcr3	Inhibition increases susceptibility to *P. infestans*	Shabab et al. (2008)
	Pip1		

The well-characterized case of the tomato PLCPs Rcr3 and its paralogue Pip1, and the inhibitors that target them, is emblematic of the complexity behind the dynamics of pathogen-derived PIs. Both Rcr3 and Pip1 are inhibited by Avr2 from *C. fulvum*, extracellular protease inhibitors with a cystatin-like domain (EPICs) from *Phytophthora infestans*, and even bacterial-derived inhibitors such as C14-inhibitor protein-1 (Cip1) (Shindo *et al.*, 2016), although only the Rcr3–Avr2 pair triggers HR. Indeed, it has been hypothesized that this is the main function of Rcr3—first acting as a decoy and co-receptor for Avr2, then triggering a downstream response with Cf-2, an immune receptor—with its paralogue Pip1, being the functional target of Avr2. This is supported by the fact that *pip1* mutants are rendered much more susceptible to *C. fulvum* in a non-Cf-2 background, while *Sprcr3* (*Solanum pimpinellifolium* Rcr3) mutants are not (Rooney *et al.*, 2005; Ilyas *et al.*, 2015).

However, when considering the role of PLCPs in immune responses (Misas‐Villamil *et al.*, 2016), it is still worth speculating what roles Rcr3 may play in basal immunity against *C. fulvum* or other pathogens. The targeting of PIs from pathogens unrelated to *C. fulvum* and that do not trigger Cf-2-related immune mechanisms (e.g. EPICs of *P. infestans*) suggests that Rcr3 constitutes a target of interest for other pathogens. Indeed, although *rcr3* mutants were also unaffected by infection with *P. syringae*, they were rendered more susceptible to *P. infestans* (Ilyas *et al.*, 2015), which has been observed prior to this (Song *et al.*, 2009). This would imply that *Sp*Rcr3 can provide basal immunity to *P. infestans*, a notion that was built upon by Kourelis and associates, where they integrate the intertwined evolutionary histories of Avr2, Rcr3, and Cf-2. It was found that although Avr2 binding and inhibition was most effective in *Sp*Rcr3, this was still suboptimal due to specific amino acid substitutions, also affecting other pathogen-derived inhibitors. One of the hypotheses put forward by the authors was that this consisted of a way of maintaining the role of Rcr3 in basal immunity in the absence of Cf-2. In this way, Rcr3 can play a role on both sides, lying in the middle ground as a co-receptor of Avr proteins and a player in basal immunity against various pathogens (Kourelis *et al.*, 2020). Conversely, there have also been naturally occurring changes in Avr2 protein sequence that resulted in the loss of Cf-2-mediated immune responses, ranging from point mutations resulting in amino acid substitutions to partial or complete deletions (Luderer *et al.*, 2002; Mesarich *et al.*, 2023). This noteworthy body of work shows how the structure and mechanisms of PIs provide valuable insights into the evolutionary history of protease inhibition mechanisms, and their relationship to plant immunity. The conservation of PIs and/or their mechanisms across taxa is of great importance, not only at the fundamental level but also when considering biotechnological applications. Another interesting example of a possible cross-kingdom conservation of a PI motif can be found in protein involved in tumors 2 (Pit2), an inhibitor of the previously discussed maize pathogen *U. maydis*. Similarly to CC9, Pit2 can inhibit apoplastic PLCPs such as CP2 and CP1A, homologues of RD21 and XCP1, hampering SA-mediated immunity and contributing to the establishment of a compatible interaction. It was also found that Pit2 carries within it a 14 amino acid motif, termed Protease Inhibitor Domain 14 (PID14), that is responsible for both interacting with and inhibiting the target PLCPs (Mueller *et al.*, 2013). In subsequent work, it was found that this very motif serves as bait for the proteases it inhibits by being cleaved from the rest of the effector and remaining bound to its target. Interestingly, this motif was found to be conserved across filamentous fungi, yeasts, and bacteria, with the latter appearing to be the ones with greatest sequence similarity. Furthermore, it was also shown that these motifs preserve their functionality, since one of these sequences from *Streptomyces* sp. was shown to successfully inhibit papain (Misas Villamil *et al*., 2019). This shows a remarkable degree of conservation across kingdoms and suggests that this mechanism of pathogenicity may also be evolutionarily conserved, although this was not elucidated. It would also be important to understand if other important pathogens employ PID14-carrying PIs as protease inhibition strategies, adding to the increasing body of knowledge on the evolutionary pressure towards PLCP inhibition across taxa.

Interestingly, XCP1 appears to be targeted by not only fungal pathogens, but also by oomycetes, as evidenced by the discovery of the cystatin-like PI from *Plasmodiophora brassicae*, the causal agent of clubroot disease in *Brassica* spp. In this work, Pérez-López *et al.* (2021) found that the apoplastic effector small secreted *P. brassicae* protein 53 (SSPbP53) interacted with and inhibited several PLCPs of Arabidopsis in the apoplast, with XCP1 appearing to be the target that sustained the greatest inhibitory effect. Interestingly, *xpc1* mutants were rendered more tolerant to *P. brassicae*, which could suggest that SSPbP53-mediated inhibition of XCP1 could be detrimental to its virulence, and that XCP1 is not a resistance factor for this biotroph specifically. In addition, *XCP1* gene expression is repressed upon *P. brassicae* infection, which could represent a possible defence mechanism by the plant in response to infection. Despite this, it is still unclear how SSPbP53 plays a role in *P. brassicae* virulence. It could be of value to inoculate Arabidopsis lines overexpressing the inhibitor construct and evaluate disease progression in that background, thus capturing a scenario where XCP1 (along with other PLCPs targeted by the effector) is potentially inhibited.

With the advent of new technological breakthroughs in the field of bioinformatics, harnessing tools to predict structures and interactions between proteases and inhibitors has become much more important, being a low stake starting point with great predictive power. For instance, Sarkar *et al.* (2023) showed robust *in silico* proof of an inhibitory interaction between *M. oryzae* rare lipoprotein A (*Mo*RlpA) and a rice CathB-like PLCP, ranging from molecular docking to 3D structure prediction and molecular dynamics simulations. However, no experimental evidence was obtained to confirm this interaction, which could have important implications for the pathogenicity of this fungus as an inhibitor of cysteine proteases. Interestingly, however, Charova *et al.* (2020) uncovered the homologue RlpA in *Rhizoctonia solani* (*Rs*RlpA) that can effectively inhibit PLCPs and interact with a CathB-like protease of *Nicotiana benthamiana*. Furthermore, *Rs*RlpA serves as a negative regulator of cell death *in vivo* and promotes virulence when expressed in the hemibiotroph *Cercospora beticola* against sugar beet, providing more evidence of RsRlpA as an important effector in biotrophy (Charova *et al.*, 2020). Indeed, bioinformatics can also be a valuable tool for *de novo* identification of PIs from pathogen secretomes, using a streamlined workflow for uncovering putative inhibitors. One example of this is the discovery of the apoplastic Cip1 inhibitor from *P. syringae*, which was found to inhibit the RD21 orthologue C14 of tomato, along with Pip1 and Rcr3, although the latter with lower affinity (Shindo *et al.*, 2016).

Very recently, and in tune with recent advances in artificial intelligence applied to protein–protein interaction, Homma *et al.* (2023, 2024) set a precedent on uncovering novel PIs from databases of pathogen secretomes using AlphaFold-Multimer. The authors screened >11 000 small-secreted peptides (SSPs) from seven different tomato pathogens for interactions with six hydrolases with roles in immune responses, with a special focus on the tomato P69B, a subtilisin-like serine protease. By applying strict protein–protein interaction criteria along with transcriptomic data, researchers identified a refined group of peptides that specifically target P69B. These peptides include SSP from *Xanthomonas perforans* (*Xp*Ssp1), extracellular protein 36 from *C. fulvum* (*Cf*Ecp36), and both trypsin inhibitor-like protein (*Fo*TIL) and secreted into xylem 16 (*Fo*Six16) from *Fusarium oxysporum*. They were confirmed to inhibit P69B at levels comparable with *Pi*EPI1, a well-known Kazal-like inhibitor of this subtilase (Homma *et al.*, 2023). This approach can significantly expedite and expand the scope of the search for novel inhibitors, due to its computing power and not requiring conventional methods of conserved domain queries. Indeed, only one of the four uncovered inhibitors carries a conserved domain, *Fo*TIL, evidence of the remarkable potential of this approach in mining new putative PIs (Homma *et al.*, 2023). Nonetheless, AlphaFold should be used with caution as many proteins do not adopt a stable 3D structure under physiological conditions. Thus, they may display multiple conformations which often allow them to interact with various partners, which is also known as multivalency. This characteristic makes it more challenging to predict their exact structure using computational tools and, consequently, their potential partners.

The notion of specific immune-related proteases as hubs of targeted inhibition by pathogen-derived PIs is not new, having previously been discussed in the context of PLCPs (Misas‐Villamil *et al.*, 2016). Indeed, RD21 was also noted to be a hub of pathogen effector targeting, including PIs (Huang and van der Hoorn, 2025). In this study, the authors note an inter-kindgom targeting of RD21 homologues across pathosystems, including pathogens such as viruses, TYLCV V2Pro (Bar-Ziv *et al.*, 2015; Liu *et al.*, 2023); bacteria, Cip1 and SDE1 (Shindo *et al.*, 2016; Clark *et al.*, 2018); oomycetes, EPIC1/2B (Kaschani *et al.*, 2010); fungi, Pit2 and *Mo*Ers1 (Mueller *et al.*, 2013; M. Liu *et al.*, 2024); and nematodes (J. Yu *et al.*, 2024). Considering the important role of RD21 in basal immunity against a plethora of pathogens, it is clear why it has become a centre point in pathogen-mediated inhibition, even though its precise role is still not clear. The subtilase P69B can also be considered a hub for pathogen-mediated inhibition, especially when considering its role in immune responses. Indeed, in addition to the PIs from the bacterial and two fungal pathogens previously mentioned, P69B is targeted by two Kazal-like PIs from *P. infestans*, EPI1 and EPI10 (Tian *et al.*, 2004, 2005). Again, this shows multiple independent events where plant pathogens converge on the same mechanism of virulence, that being the inhibition of an important immune-related protease. Relating P69B inhibition to its functions in immunity, it becomes apparent why this possible pressure towards inhibition arose in the first place. It was recently found that P69B can act as a trigger of PTI by cleaving a *P. infestans*-derived peptide, PC2, with its inhibition having been linked to a dampening of this response (Wang *et al.*, 2021). Furthermore, P69B plays a key role in Rcr3 activation in the apoplast via proteolysis, ensuring its activation when Rcr3 autocatalytic activation is impossible due to either inhibition by Avr2 or the physicochemical conditions of the apoplast (Paulus *et al.*, 2020).

## Protease (inhibitor) engineering as a tool for crop protection

Considering the prolific roles PIs play in either suppression (pathogen-derived inhibitors), regulation (self-inhibitors), or intensification (host-derived inhibitors) of plant defence mechanisms, the hypothesis of harnessing them towards improving plant fitness against pathogens and pests arises. To achieve this, it is imperative that knowledge regarding proteases, PIs, and their interactions is continuously obtained, whether it be structural, biochemical, or functional (van Wyk *et al.*, 2016). From specific amino acid residues that are crucial for interactions to differential binding among orthologues of the same protease (Kourelis *et al.*, 2020) or PI, this variability provides fertile grounds for promising new ways of engineering towards increased plant resilience.

Although protein engineering may be regarded as a more enticing approach to plant improvement against biotic stress, more conventional approaches of gene overexpression or pyramiding can also prove to be viable options. Various reports have been made throughout the years concerning endogenous (Liu *et al.*, 2021) or heterologous (Senthilkumar *et al.*, 2010) expression of PI genes conferring enhanced tolerance to pathogens and pests, as well as pyramiding of multiple genes (Senthilkumar *et al.*, 2010) (reviewed in Pandey *et al.*, 2022) (see ‘Inhibition of pest/pathogen proteases by host-derived inhibitors’). Many of these methodologies could be considered in conjunction with protein engineering, although they inevitably fall under the umbrella of transgenics, which could hinder their deployment in regions that do not allow their cultivation. However, in places where transgenic crops are currently greenlit for cultivation, such as North and South America and certain countries in Asia, the prospect of deploying crops transformed with PI genes under this framework appears to be more feasible. Nevertheless, the advent of new genomic techniques allows for cisgenics-based approaches, enabling the use of endogenous PI genes for genetic engineering or mutagenesis-based techniques, broadening the plant breeder’s toolkit (Giudice *et al.*, 2021).

The tomato multicystatin (*Sl*CYS8) has served as a model of sorts to develop new protein engineering approaches, in part due to the prevalence of positively selected, hypervariable sites in its eighth inhibitory unit. Specific hypervariable residues were identified for mutagenesis via phylogenetic analysis and *in silico* determination of protein–protein interaction strength, selecting Proline 2 (Pro2) as a candidate (Kiggundu *et al.*, 2006). Indeed, substitutions of Pro2 by different hydrophobic residues (Pro2Phe, Pro2Ile, Pro2Leu, and Pro2Tyr) rendered *Sl*CYS8 much more effective in inhibiting cysteine proteases, while making plants more resilient to herbivory (Goulet *et al.*, 2008). Hydrophobic residues have in fact been heavily implicated in protein–protein interactions (Rego *et al.*, 2021), including plant PIs (Kojima *et al.*, 1996), suggesting a particular role for these residues in protease–inhibitor interactions. Beyond targeted mutagenesis, other approaches have been proposed for inhibitor engineering, such as the development of chimeric proteins with domains from homologues with different degrees of activity, termed loop replacement design. In their work, Tremblay and associates fused inhibitory loops of CYS8 homologues from *Physcomitrella patens* and *Solanum tuberosum*, which have higher inhibitory potentials, to *Sl*CYS8. Consequently, these chimeras showed significantly higher inhibitory potential than both wild-type *Sl*CYS8 and its previously discussed single amino acid substitution variants, providing a new avenue for effective cystatin engineering (Tremblay *et al.*, 2022). It is tempting to speculate whether this approach could be extended to other classes of protease inhibitors, given that this technique may be more effective than single amino acid substitutions.

Another potential PI engineering approach may lie in cyclic peptides, which have already been shown to be effective in controlling some pests. Saikhedkar *et al.* (2019) generated cyclic peptides from the reactive centre loops of two Pin-II PIs fused to a benzene scaffold, which showed a 10-fold increase in protease inhibition capacity when compared with their native, non-cyclic variants, along with strong herbivory deterrence. Although more proofs of concept are still required, cyclic peptides could also present an interesting new alleyway in modulating protease activity in plant breeding.

It is important to note the ability of pathogens and pests to swiftly overcome these modifications, either by modulating protease gene expression or by expressing inhibitor-insensitive proteases (Pandey *et al.*, 2022). Dealing with this question could involve the introduction of more redundancy, by harnessing either various PIs simultaneously or more multifunctional inhibitors.

The bioengineering of proteins in the context of PI–protease interactions can also be directed towards proteases, considering that protease affinity for inhibitors can also be altered precisely. For some cases, it could be of interest to decrease protease sensitivity to inhibition, to restore their functionality in the context of an infection. To this end, Schuster *et al.* (2024) engineered Pip1 via the mutation of two residues, to achieve insensitivity to protease inhibitor EpiC2B from *P. infestans*. It was demonstrated that the EpiC2B-insensitive Pip1 (ePip1) increased immunity against *P. infestans*, when compared with wild-type Pip1. Kourelis *et al.* (2020) identified four critical residues in tomato Rcr3 required for Avr2 inhibition and subsequent Cf-2-mediated signalling. The residues consisted of a negatively charged residue at position 148, an uncharged small residue at position 151, an Asn residue at position 194, and a positively charged residue at position 284. The substitutions D194N or A284R for C14 and D284R for CYP3, PLCPs which are not naturally inhibited by Avr2, were sufficient to establish Avr2 inhibition (Kourelis *et al.*, 2020). Knowing that these key residues are responsible for increasing the affinity of Avr2, the authors engineered two homologues of tomato Rcr3, *Solanum melongena* Rcr3 (aubergine; *Sm*Rcr3) and *N. benthamiana* Rcr3 (*Nb*Rcr3), to trigger Avr2/Cf-2-dependent immunity, since these proteases could not trigger the response naturally. It was found that a D244P mutation in *Sm*Rcr3 triggered HR upon co-expression with Cf-2 and Avr2. On the other hand, a G194N substitution and an insertion of a DPS motif in *Nb*Rcr3 resulted in Avr2/Cf-2-dependent HR (Kourelis *et al.*, 2024). While the G194N substitution was already predicted to increase binding to Avr2, the tripeptide DPS, omitted from *Nb*Rcr3 but present in other *Solanaceae* Rcr3 homologues, was found to be crucial for triggering HR. This fine-tuning of protease sensitivity to inhibitors, only made possible by in-depth knowledge regarding their interaction mechanisms, is essential for the bioengineering of novel proteases. In addition, since these approaches are based on point mutations, the potential for them to be more widely accepted and implemented could be higher, particularly in regions where genetically engineered crops are still met with some pushback from policymakers and the general population alike. Somewhat similarly to inhibitor engineering, this form of protein engineering could fall short of achieving meaningful impact depending on the chosen target, as it represents a highly specific form of plant improvement.

Currently, the prediction of SSPs involved in protease inhibition is possible with the aid of *in silico* tools, namely AlphaFold-Multimer (Homma *et al.*, 2023, 2024). It is also tempting to hypothesize how these tools would aid in the development of new artificially designed PIs, which could have the specificity or broadness of action desired. Very recently, a novel molecule was developed based on the stereochemistry of the interaction between the *M. oryzae* protease inhibitor *Mo*Ers1 and the rice *Os*RD21, also assisted by more advanced bioinformatic tools. Through molecular docking analysis, it was predicted that the interface of *Mo*Ers1 for interaction was rich in hydrophobic amino acid residues, with a geometry that would favour the binding of more flexible molecules. Among those molecules was FY21001, a diphenyl ether ester which bound *Mo*Ers1 in its interaction surface and successfully blocked its inhibitory function, rescuing RD21-mediated immunity (M. Liu *et al.*, 2024). To our knowledge, this was the first time a molecule was found to inhibit protease inhibition in plants, leading to restricted pathogen growth, setting a precedent for new drug discovery approaches for modulating protease inhibition *in vivo*.

## Conclusion

The body of knowledge on the role of proteases and their inhibitory dynamics is ever increasing, not only enabling further research but also expanding the number of biotechnological approaches for plant improvement against biotic stress. Recent steps have been taken in the direction of identifying hubs of protease inhibition, with RD21 (and PLCPs more generally) and P69B already noted to have this feature. In addition, and considering new evidence, we note that XCP1 and CathB, other PLCPs, may also be hubs for protease inhibition, although it would be interesting to investigate if more inhibitors do indeed target these PLCPs. Conversely, we provide evidence of the prolific roles of serpins in self-regulation of proteolysis, representing a conserved mechanism with tremendous potential for biotic stress amelioration. However, many questions and prospects for future research still exist.

At a fundamental level, much is still unknown about the specific roles of proteases in plant immunity. Could their mechanisms be direct proteolysis of pathogen tissues, or could they have other roles? While many PLCPs act as pro-cell death proteases, from some metacaspases such as AtMC1 to XCP1, Rcr3 is a flagship of proteases as immune receptors, with its proteolytic activity *per se* having received less attention—which other proteases have similar roles?

We also identified a significant gap in knowledge regarding the role of plant-derived PIs as a mechanism of immunity against pathogens. As it stands, much of the research focuses on insect pests, albeit with promising results as a plant protection measure. However, when it comes to microbial pathogens, this gap in knowledge is significantly heightened, despite the topic having garnered decent attention from the scientific community (Figaj *et al.*, 2019; Lee Erickson and Schuster, 2024). Perhaps a more in-depth knowledge of pathogen protease diversity and mechanisms is first required, although with the advent of tools such as AlphaFold-Multimer, the uncovering of new protease–PI pairs will surely be expedited. A comprehensive proteome analysis that examines both pathogen proteins and the apoplastic proteome could significantly enhance our understanding of plant immunity mechanisms. By capturing the dynamic interplay between the secreted proteins of the pathogen and the apoplastic proteins of the host, researchers can pinpoint critical interactions—such as protease and PI pairings—that are essential for defence.

As for the biotechnological prospects, the notion of hubs of inhibition may be of importance once again, as the targeting of a single protease by different inhibitors could be exploited in this sense. If more information regarding protease–PI interaction were elucidated, would it be possible to engineer a protease that is insensitive to all its known inhibitors, without losing function? Further research is needed to determine whether broad-spectrum resistance can be effectively engineered into plants. In contrast to proteases, PI engineering presents great biotechnological potential as a biocontrol measure. Are there more broad-spectrum PIs like APIP4 from rice?
